# The Evolution of Anorexia Nervosa in Singapore: A 30-year Demographic Analysis

**DOI:** 10.1192/j.eurpsy.2024.485

**Published:** 2024-08-27

**Authors:** H. Y. Lee, V. K. H. Wong

**Affiliations:** Psychiatry, Singapore General Hospital, Singapore, Singapore

## Abstract

**Introduction:**

New prevalence and time trend data from various Asian countries show that Eating Disorders (ED) are increasingly common in Asia. (Youl-Ri Kim. Int J Eat Disord. Dec 2020). A recent study estimating the prevalence of ED in Singapore found an alarming 6.2% screened positive for a clinical ED diagnosis, 19.5% were screened to be at high risk, and estimated the point prevalence of Anorexia Nervosa (AN) to be 0.9%. (Chua SN et al. Int J Eat Disord. Jan 2021).

The ED unit in Singapore General Hospital (SGH) was set up in 2003 as a National Treatment Programme for patients with ED.

Two local studies have been published to date on the demographics and clinical profile of patients with AN. The first study examined 126 patients from 1994 – 2002 (HY Lee et al. Singapore Med J 2005; 46(6): 275-281). The second study reported on 271 cases from our SGH ED unit from 2003-2010 (Kuek et al. SIngapore Med J 2015; 56(6): 324-328). There have been no further studies in the last decade.

**Objectives:**

1. Study the demographics and clinical profile of patients who presented with AN to our ED unit from 2011-2022
2. Compare our data with the 2 previous studies and examine for any changes and trends in the past 30 years.

**Methods:**

We conducted a review of the ED unit new case registry at SGH from 2011-2022. A total of 910 patients were diagnosed with AN at presentation. The data was analysed with approval from the hospital instituitional review board.

**Results:**

A total of 910 cases presented with AN over 12 years. Comparing with the 2 previous studies, the number of new cases each year has continued to increase from <15 in the 1990s to hit a peak of 109 per year in 2022. 94% were females, with a mean presenting age of 19. 79.2% were Chinese, 5.2% were Indians and 2.9% were of Malay ethnicity. The Malay population continue to be under-represented whereas other ethnic groups continue to be over-represented, increasing from 3.2% to 7% in the previous studies to 11.1%. Referrals were mainly from tertiary healthcare intuitions accounting for 41.4% of cases. Self-referrals have decreased over the last decade whereas referrals from primary care has increased. The mean presenting body mass index (BMI) was 15.9. Compared to a previous study, there was a significant increase in presenting BMI (15.9+/- 0.78 vs 14.4 +/- 1.77, p value 0.0074).

**Conclusions:**

The number of new cases of AN has seen an almost 10-fold increase in the last 30years. The Malay ethnicity continues to be under-represented – more research is needed if they are somehow culturally protected or if they are not coming forth for treatment. Majority of referrals are from tertiary healthcare institutions but referrals from primary care have increased, reflecting a possible increase in awareness amongst primary care doctors. The mean presenting BMI has increased – hopefully reflecting an increase in ED awareness such that patients are coming forward earlier for treatment.

**Disclosure of Interest:**

None Declared

